# Oxidative stress and food as medicine

**DOI:** 10.3389/fnut.2024.1394632

**Published:** 2024-08-23

**Authors:** DuWayne A. Carlson, Cheryl True, Christopher G. Wilson

**Affiliations:** ^1^Community Hospital of Grand Junction, Grand Junction, CO, United States; ^2^Genesis Health System, Davenport, IA, United States; ^3^Department of Physiology, Loma Linda University, Loma Linda, CA, United States

**Keywords:** whole food antioxidants, antioxidants, pro-oxidants, triple oxidant sink, antioxidant lifestyle, oxidative stress, redox balance, processed foods

## Abstract

There has been a sea of change in our understanding of the contribution of food to both our well-being and disease states. When one addresses “food as medicine,” the concept of oxidative stress needs to be included. This review interconnects the basic science findings of oxidative stress and redox balance with the medicinal use of food, emphasizing optimization of the redox balance. To better illustrate the impacts of oxidative stress, the concept of the “triple oxidant sink” is introduced as a theoretical gauge of redox balance. Utilizing the concept, the true importance of dietary and lifestyle factors can be emphasized, including the limitations of supplements or a handful of “superfoods,” if the remainder of the factors are pro-oxidant. The effects of a whole plant food diet compared with those of dietary supplements, processed foods, animal based nutrients, or additional lifestyle factors can be visually demonstrated with this concept. This paper provides an overview of the process, acknowledging that food is not the only mechanism for balancing the redox status, but one that can be strategically used to dramatically improve the oxidative state, and thus should be used as medicine.

## 1 Introduction

Oxidative stress has been defined by the National Institutes of Health (NIH) in the U.S. as “a condition that may occur when there are too many unstable molecules called free radicals in the body and not enough antioxidants to get rid of them. This can lead to cell and tissue damage.” (1) There is a growing body of evidence that oxidative stress is heavily involved with, and responsible for, disease onset and progression, but can potentially be mitigated—at least partially—by whole plant foods. Whole plant foods can be defined as minimally processed plant foods such as fruits, vegetables, grains, legumes, nuts and seeds, and herbs and spices (2). Understanding the interplay of nutrients supplied by whole plant foods and oxidative stress pathways, both systemically and at the cellular level, is a frontier that is still only partially understood. In this paper we explore these important functions.

### 1.1 Oxidative stress/redox imbalance

Oxidative stress creates an imbalance of pro-and antioxidants in an organism which has both intrinsic and extrinsic compensatory mechanisms driving homeostasis (3). The most recognized pro-oxidants include free radicals and hydrogen peroxide (H_2_O_2_) (4). An excess of pro-oxidants, especially free radicals, contributes to cellular injury. As free radicals, including reactive oxygen species (ROS) and reactive nitrogen species (RNS) are extremely reactive and cause chain reactions impacting many cellular functions, extensive cellular injury can result. Cell wall phospholipids, DNA, enzymes, other proteins, and other cellular components can be damaged by the free radicals and altered by the other pro-oxidants. The redox (reduction–oxidation) imbalance generated is one of the originators of the pro-inflammatory cytokine cascades producing the characteristic IL-1β, 4, 6, 8, 18, HIF, and TNF-ɑ (4). Redox imbalance also inhibits progenitor cells (5, 6). Oxidative stress also induces epigenetic alterations in DNA methylation, histones, and non-transcriptional miRNAs which alter transcription, translation, and ultimately, cellular physiology which leads to disease states (7, 8). Cellular homeostasis is then disrupted and physiology is altered by the oxidative stress.

Oxidative stress is strongly and broadly associated with many and, possibly, *all* chronic diseases. There are those that are tightly linked to oxidative stress including type 2 diabetes (9), heart disease (10–12), and cancer (13, 14). Other associations noted in the literature include congenital defects (not from genetic causes) (15), autism (16), ADHD (17), neurodegenerative disorders (18), rheumatologic diseases (19), psychiatric diagnoses (20), kidney diseases (21), liver diseases (22), asthma/COPD and idiopathic pulmonary fibrosis (23). Even less common conditions have an oxidative stress link including chronic regional pain syndrome-1 (24), sickle cell crisis (25), exacerbations of muscular dystrophy (26), and liposomal storage diseases (27). It is unclear if there are *any* diseases or medical conditions that would be completely independent of oxidative stress.

All organisms appear to have intrinsic defense mechanisms to combat the redox imbalance of the pro-oxidants and antioxidants causing oxidative stress. Endogenous/intrinsic enzymatic antioxidants (host) neutralize free radicals like superoxide dismutases and others. Other enzymes break down pro-oxidants including hydrogen peroxide such as peroxidases and catalase (20). These enzymes are located throughout the cell and provide a surveillance system to address oxidative stress.

Intrinsic non-enzymatic antioxidant defense is based on free radical scavengers (20). Glutathione is thought to be one of the most important non-enzymatic antioxidants (28). Albumin, the most common circulating protein in the body, serves as an abundant and important roving free radical scavenger, utilizing disulfide bonds to neutralize the dangerous free radicals (29, 30). Estrogen is another powerful antioxidant through both direct scavenging and stimulating increased expression of endogenous antioxidant enzymes (31, 32).

### 1.2 Whole plant antioxidants

Extrinsic methods of oxidative stress neutralization come from dietary sources. There are three main categories: free radical scavengers, immunomodulators, and stimulators of the ARE (antioxidant related element, or upregulation of the production of antioxidants and antioxidant enzymes) (33, 34). Dietary sources of free radical scavengers have characteristically been compared by their ORAC value—oxygen radical absorption capacity (35). Each ORAC unit is defined as having the capacity to absorb one free radical. Thus, the higher the ORAC value, the greater the ability to absorb oxygen or nitrogen-based free radicals.

Immunomodulators are molecules that downregulate the production of downstream inflammatory cytokines, leukotrienes, complement, and prostaglandins (4). The ARE (antioxidant related element) is the section of DNA that encodes antioxidant enzymes and free radical scavengers (3), and is often stimulated through the enzyme NF-E2-related factor 2 (Nrf2) (36). Immunomodulation and/or ARE stimulation is found from sulforaphane, found in broccoli and other cruciferous vegetables (34), quercetin, found in many fruits and vegetables, vitamin D and B12, and others. Many of the antioxidants supplied by whole plant foods act as free radical scavengers, immunomodulators, and ARE stimulants which include polyphenols and flavonoids (4).

Though not technically classified as an immunomodulator, the gut microbiome is another source of immunomodulation for oxidative stress. Short chain fatty acids (especially butyrate) formed by pro-health gut bacteria have been shown to serve a significant role in lowering oxidative stress (37). In addition, it has been found that many antioxidants found in dietary sources, especially polyphenols, are bound to fiber. These fiber bound polyphenols are only accessible to the organism when the appropriate gut microbes break down the fiber and release the antioxidants (38). Antioxidants and fiber, on the background of a supportive gut microbiome, appear to be a crucial/critical component for deactivating free radicals and other pro-oxidants. This allows for suppression or extinguishing the downstream cytokines, other inflammatory molecules, boosting endogenous antioxidant enzymes, and in general restoring the redox balance of the entire organism.

While there are low levels of antioxidants in animal products residual in the tissue from their plant food ingestion, the concentration of antioxidants is exponentially greater in whole plants, relative to intake volume (39). In addition, fiber occurs exclusively in plant-derived foods, not animal products.

The immunomodulatory effects of antioxidants typically act by suppressing the production and effect of inflammatory cytokines (IL-6, IL-1β, IL-4, IL-18, TNF-α, NFκB, etc.) and increasing anti-inflammatory cytokines (Nrf-2, IL-10, and others) production (4, 33, 40). In addition there is inhibition of lipid peroxidation, oxidation of LDL, and leukotriene and complement production (33).

Sulforaphanes (from cruciferous vegetables) are among the most potent immunomodulators (34), and have been shown to have multiple effects including:

1. Activation of Nrf2-mediated phase II enzymes. Upregulating Nrf2 induces the antioxidant response element DNA sequence transcription, encoding for increased endogenous antioxidant enzymes (34, 41, 42). Sulforaphane may be the most potent natural stimulator of Nrf2 (43).
2. Downregulation of NfκB which plays an important role in the production of the oxidative stress cytokines (44).
3. Upregulation of glutathione production (an endogenous non-enzymatic antioxidant) (45).
4. Increased production of heat shock proteins further promoting cell stability (46).
5. Decrease in secretory leukocyte protease inhibitor which inhibits proteases like cathepsin G and neutrophil elastase (which are both associated with lung damage) (47, 48).

Quercetin, a flavonoid, is abundant in fruits and vegetables and has been shown to suppress NLRP3 inflammasomes with a subsequent downstream suppression of IL-1β, and IL-18 (49). Additionally, glutamine, found in sorghum, walnuts, kale, kidney beans, and red cabbage has been shown to decrease inflammatory markers IL-1β, TNF-α and hs-CRP in COVID patients (50). These compounds, so prevalent in plants, provide a significant anti-inflammatory substrate to bolster antioxidant reserves.

Fiber, in addition to ferrying bound antioxidants, produces short chain fatty acids, including butyrate, when in the presence of health promoting gut microorganisms. Butyrate has been shown to significantly lower oxidative stress (37) and decrease TNF-α mediated immune responses including mitochondria derived inflammasomes such as NLRP3 (51). Additionally, it has been shown to have an anti-inflammatory effect on the bone marrow derived macrophages strongly decreasing IL-6 and inducible nitric oxide (iNO) in a dose-dependent fashion (51, 52). The mitochondrial induced oxidative stress is also decreased through communication with a healthy gut microbiome (51).

Specific foods that have been studied include spices, which have a significant effect on the gut microbiome stimulating the growth of beneficial bacteria and inhibiting the growth of the pathogens (53–55). A number of spices have also been shown to be immunomodulators, decreasing TNF-ɑ, IL-1ɑ, and decreasing DNA damage (56). Additionally, herbs and spices have very high ORAC values giving very good free radical scavenging ability.

Garlic, in different models, has been shown to decrease phosphorylation of ERK1/2 to induce inflammation in adipocytes to LPS stimulation. Decreased histamine release by basophils has been noted in another model of garlic supplementation. Decreases in IL-6, TNF-α, and NK-κB have also been shown (40). There is a growing body of literature focused on the therapeutic effects of garlic in many different diseases as reviewed by Majewski (57). Though there is some debate about clinical efficacy of garlic as a nutraceutical rather than a whole plant food (58).

Fruits and vegetables influence oxidative stress through a host of bioactive compounds including the following examples. Vitamin C, a strong free radical scavenger, protects cells from inflammatory dysfunction and neurodegenerative diseases (33). Vitamin E is a fat soluble chain breaking antioxidant protecting DNA and polyunsaturated fatty acids. Polyphenols improve gut mucosal immunity and inflammation, promote IL-10 generation (anti-inflammatory IL), along with many other antioxidant functions. Flavonoids possess antioxidant, anticancer, and antimutagenic properties (33). One of the carotenoids, lycopene, is a powerful free radical scavenger, in low concentrations. Lycopene stimulates Nrf2 as well as the rest of the ARE, stimulating endogenous antioxidant enzyme production (59). Salicylic acid, sometimes known as vitamin S, is usually known for its inhibition of cyclo-oxygenase. Though this is a part of its action in suppressing prostaglandin formation, other activities include MAPK and NfKb inhibition, binding of iron leading to prevention of lipid peroxidation, and appears to inhibit production of inducible nitric oxide (pro-inflammatory) (60).

### 1.3 Antioxidant supplements vs. plant derived antioxidants

When considering diseases or conditions exacerbated by oxidative stress, or an overabundance of pro-oxidants, the obvious solution would seem to be supplementing with antioxidants to reverse the disease process (43, 61–64). Physiologic doses of exogenous antioxidants are required to maintain or re-establish redox homeostasis (65). Attempts to utilize isolated components in the form of antioxidant supplements have often not produced the desired result of disease prevention or reduction, and at times were actually harmful (66–71). For example, manufactured antioxidant supplements for occlusive heart disease has been harmful rather than helpful (71–73). However, there is data showing that consuming whole plant foods which naturally contain antioxidants are successful in disease modification (72, 74). So the synergistic effects of eating whole plant foods outweighs the current trend of providing an isolated supplement in pill form to serve as stand-alone drug.

A working hypothesis is that there is synergy between the antioxidants contained within each whole plant likely indicating that isolated antioxidants do not fulfill their characteristic role without synergistic partners. Antioxidant supplements do not provide physiological benefit in higher doses (75–77). The studies performed with antioxidant supplementation do not take into account the usual low concentration of nutrients as well as the synergistic actions of the phytochemicals and other nutrients. This could explain the variable and almost always disappointing results of most human supplementation studies (65). It is also likely, though not studied to our knowledge, that fiber along with the pro-health gut microbiome acts synergistically with whole plant derived antioxidants to multiply the antioxidant effect.

Thus, returning to the hypothesis that food is medicine, it could be further argued that the ideal dietary intake of whole plant foods combined with a healthy gut microbiome contribute to the ability of an organism to better manage the oxidative stress contributing to disease states by optimizing the antioxidant capacity of the organism (43, 61, 62, 64, 65, 78). These exogenous, plant derived antioxidants would serve as free radical scavengers and immunomodulators, as they have accomplished in the chronic inflammatory diseases of diabetes and heart disease.

## 2 Processed foods

Altering plant-foods from their original forms is not a new concept, as humans have preserved foods through methods such as cooking, drying, salting, fermenting and freezing for centuries. Modern introduction of food processing strips away or extracts components of the whole food, transforming the original into a manufactured “food-like” substance, something that is potentially unrecognizable to the organism or its gut microbes. Altering whole plant foods potentially removes fiber, and destroys antioxidants, diminishing the nutritional value of the final products from a perspective of oxidative stress reduction. Processing of foods can be done to varying degrees, such as refining grains to create flour. If the whole grain is used, the flour may retain many of the benefits of the original, but when a grain is separated into component parts of endosperm, germ and bran, and only the endosperm portion is used to create flour, nutrients and fiber are stripped away. Other examples of processing include removing sugar from beets, or extracting oil from peanuts. Many of the antioxidant and trace elements obtained from fruits and vegetables can be found in the skin which is typically removed in the course of processing, with much of the fiber. The processed food is left with calories, but without antioxidants to reverse the oxidative stress induced by manufacturing of ATP. During electron transport a 1–5% rate of errors occurs in transferring the electrons between cytochromes causing formation of a superoxide free radical, most commonly at cytochrome I and III. When the organism experiences greater stress, this error rate and additional pro-oxidant production in the mitochondria increases significantly (22). In a redox balanced organism there is sufficient superoxide dismutase (SOD) and other free radical scavengers to neutralize the impact of errors in electron transfer so they do not cause ongoing problems in management of free radicals. But when the supply of antioxidants has been depleted injury can occur. Thus, foods with calories but without antioxidants push an organism’s normal physiology towards a pro-oxidant, pathophysiological state that can result in cellular and organ damage.

## 3 Animal based foods

Energy production through metabolism of the fats, proteins, and carbohydrates contained within ingested animal products (which lack fiber and significant amounts of antioxidants) creates a pro-oxidant environment, similar to what occurs after ingestion of processed foods (39). Unfortunately, with regular consumption of animal products, there are deleterious alterations to the gut microbiome and other mechanisms in place that increase oxidative stress in the consuming human. Examples include the production of TMAO [trimethylamine N-oxide (79)], higher concentration of branched chain amino acids (80), contamination of the meat/fish with PCBs (81), and harmful heavy metals such as lead and mercury (14), in addition to other toxins that contribute to a heavily pro-oxidant state.

## 4 Non-dietary lifestyle factors influencing oxidative state

There are a host of other non-dietary factors that positively or negatively affect one’s oxidative state. Positive factors include, but are not limited to exercise (82), sleep (83), hydration (84), vitamin D level and mode of delivery (85), and loving relationships (86, 87). Negative lifestyle choices influence levels of toxin intake through smoking (88) and ethanol use (89), as well as prescription drug use or other substances activating the liver’s P-450 enzymes (22), psychological stress (90), severe life stressors (91), fasting (92), and other mild stressors (hormetic stress) which induce vitagenes resulting in increased endogenous antioxidants, as well as others (93).

**Figure 1:**
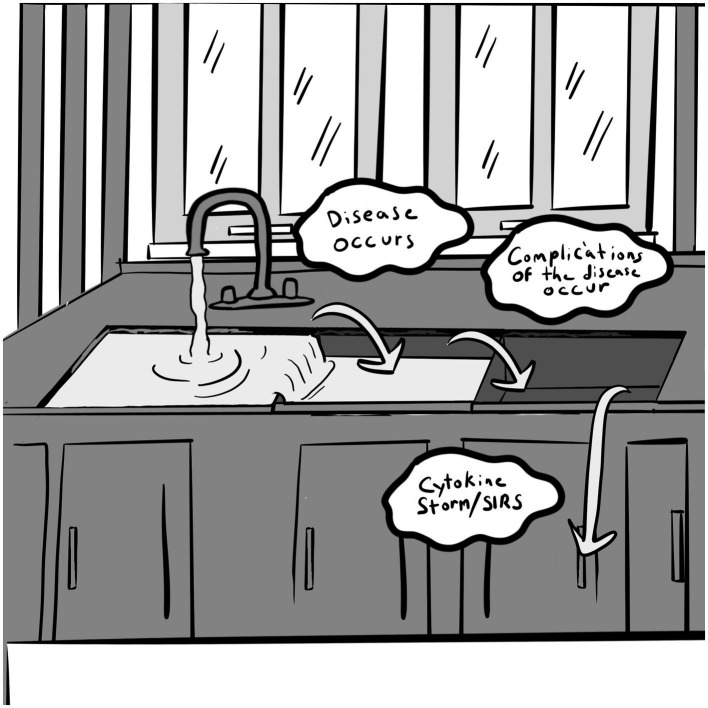
Triple Oxidant Sink. Reproduced with permission from IJDRP, licensed under CC BY-NC-ND 4.0.

We propose a “Triple Oxidant Sink” framework for addressing oxidative stress that uses an analogy of a triple basin sink with a faucet or inlet that fills one sink with water (78) (Figure 1). In this model, water represents oxidants that are produced or provided to an organism. These oxidants include free radicals of reactive oxygen species (ROS) and reactive nitrogen species (RNS), pro-oxidants of H_2_O_2_ and others. The triple oxidant sink theory has previously been described (78). Using the sink analogy, the sinks may all start at empty or low levels—representing a balanced and healthy organism. If there is a small amount of oxidant introduced, the sink can contain the water/oxidant easily, especially if the drain is functional. But if the drain is not working, and either the slow trickle continues indefinitely or the water inflow volume is increased, the first sink may fill and then start to overflow into the second. In this analogy, when the water flows into the second sink, disease occurs. The disease that develops depends upon genetic predisposition, much like type 2 diabetes or Parkinson’s disease. If the water overflow continues into the second sink and it does not drain, the second sink fills and overflows into the third sink. At this point complications of disease occur and ramify. Various factors influence how quickly the sinks fill, for example, the depth, size of the drains, and the amount of water flowing into the system. In the organism’s case, the depth of the first sink—where the oxidants are dumped—is determined by the endogenous antioxidant capacity (enzymatic and non-enzymatic) and the available exogenous antioxidant capacity. This endogenous level can differ between individuals based on the amount of genetically-determined antioxidant capacity. The oxidative state, at any given time, may be different based on variables which affect redox balance including the factors mentioned above affecting the oxidative state.

When a severe stressor presents in the form of infection (such as COVID-19), severe trauma, or life threatening sepsis, the oxidative sinks fill significantly. If the sinks are already fairly full of oxidants and a new oxidative stressor fills each succeeding sink, the likely result is an overflow of the sinks onto the floor. The organism is overwhelmed with pro-oxidants that cannot be fully eliminated and a cytokine storm ensues and the organism may end up on the floor as well!

This concept may explain why those with comorbidities have poorer outcomes with COVID-19 and with trauma. The prefilled oxidant sinks are overwhelmed with the inflammatory fallout of the infection or trauma and severe consequences ensue with acute respiratory distress syndrome (ARDS), multiple organ dysfunction syndrome (MODS), and systemic inflammatory response syndrome (SIRS). It also could explain differential outcomes in both COVID-19, trauma, and other oxidatively stressing occurrences. Similar age patients may have totally different outcomes from the infection or similarly severe traumatic event. This may even explain why a late teen-aged college student could develop SIRS when the expected outcome would be benign due to their relative youth. Picture the quintessential college student who stays up multiple nights in a row studying for finals, eating only low-quality junk food and drinking only caffeinated sodas. Then they go on Spring Break and experience additional lack of sleep, significant alcohol (and possibly other pro-oxidant substance) intake along with a continued low quality diet and poor healthy fluid intake. The gut microbiome is shifted to an unbalanced, compromised state, leading to oxidant sink filling. An untimely exposure to COVID-19 could potentially result in severe sickness and even SIRS. In this situation, lifestyle factors compound the impact of infection to compromise redox balance and resilience.

A similar inflammatory situation occurs when a patient undergoes surgery. There is an inevitable increase in oxidative stress from the tissue injury during surgery leading to activation of IL-33 with subsequent mast cell activation. There are pro-oxidants that are added from the medications used for anesthesia. If this triple oxidant sink theory is correct, in a setting of a high baseline oxidative state adding to this additional stress, complications of surgery would be expected to skyrocket.

The goal is to *empty the oxidative sink* so that disease does not occur due to a redox imbalance and then, if another oxidative stressor should occur, the oxidative sink has capacity to stem an overflow and prevent the cytokine storm seen in severe COVID-19, sepsis, and trauma. Questions about this theoretical model include: how fast could the redox state be changed in an ideal situation? Is it possible to reverse the systemic inflammatory response by emptying the sinks? Is it possible to reverse the diseases and the complications of disease by emptying their respective sinks if permanent damage has not occurred? Obviously an antioxidant based whole plant food diet is not the only factor in helping achieve redox balance, but it is a major factor that can be altered in one’s lifestyle (78). More research is needed to address these questions and provide evidence for our model.

The majority of known mechanisms for determination of health and longevity involve oxidative stress and the improvement of the organism’s redox balance. These mechanisms include FGF21 (94, 95), mTOR signaling (96), telomere length (97, 98), TMAO (79), increased proportion of branched chain amino acids (80), and others.

## 5 Conclusion

Based on the evidence we have discussed in this review, oxidative stress appears to be a major contributor to disease processes. Whole plant food derived antioxidants and fiber (in the background of a healthy gut microbiome) serve as significant mediators in the ability of the body to maintain redox balance. Thus, using whole plant foods as medicine along with maximizing positive lifestyle behaviors, and minimizing foods and activities depleting antioxidant reserves should lead to a balanced redox state. When redox balance is achieved, oxidative stress related diseases can be avoided, ameliorated, and possibly reversed. There is still a need for research to better understand how diet and lifestyle impact redox balance. Areas of research focusing on the triple oxidant sink and food as medicine could include redox balancing in infection, such as COVID-19 and other viral or bacteria-induced illnesses. Trauma and ICU settings have severe oxidatively stressed patients who would likely benefit greatly from redox balancing with appropriate medicinal whole plant foods. Almost every patient entering an emergency department (ED) has redox imbalance problems. Would it be reasonable to treat a patient coming into the ED with lung disease and COPD exacerbation with a breathing treatment and whole plant antioxidants and fiber (rather than a cheeseburger, fries, and a milkshake)? How might outcomes change if patients receiving a stent for a myocardial infarction were introduced to a whole plant food diet? We suggest that understanding the value and interplay of redox balancing, using food medicinally and incorporating other antioxidant behaviors, is key for optimizing nutrition and health.

## Author contributions

DC: Writing – review & editing, Writing – original draft. CT: Writing – review & editing. CW: Writing – review & editing.

## References

[1] NCI Dictionary of Cancer Terms-NCI (2011). Definition of oxidative stress. Available at: https://www.cancer.gov/publications/dictionaries/cancer-terms/def/oxidative-stress (Accessed July 19, 2023).

[2] Ostfeld RJ. Definition of a plant-based diet and overview of this special issue. J Geriatr Cardiol. (2017) 14:315. doi: 10.11909/j.issn.1671-5411.2017.05.008, PMID: 28630607 PMC5466934

[3] Tan BL Norhaizan ME Liew WPP Sulaiman RH. Antioxidant and oxidative stress: a mutual interplay in age-related diseases. Front Pharmacol. (2018) 9:1162. doi: 10.3389/fphar.2018.01162, PMID: 30405405 PMC6204759

[4] Pisoschi AM Iordache F Stanca L Gajaila I Ghimpeteanu OM Geicu OI. Antioxidant, anti-inflammatory, and immunomodulatory roles of nonvitamin antioxidants in anti-SARS-CoV-2 therapy. J Med Chem. (2022) 65:12562–93. doi: 10.1021/acs.jmedchem.2c01134, PMID: 36136726

[5] Bai X Lu D Bai J Zheng H Ke ZY Li XM. Oxidative stress inhibits osteoblastic differentiation of bone cells by ERK and NF-kappaB. Biochem Biophys Res Commun. (2004) 314:197–207. doi: 10.1016/j.bbrc.2003.12.073, PMID: 14715266

[6] Lee J Cho YS Jung H Choi I. Pharmacological regulation of oxidative stress in stem cells. Oxidative Med Cell Longev. (2018) 2018:1–13. doi: 10.1155/2018/4081890 PMC6186346 30363995

[7] García-Guede Á Vera O Ibáñez-de-Caceres I. When oxidative stress meets epigenetics: implications in Cancer development. Antioxidants. (2020) 9:468. doi: 10.3390/antiox9060468, PMID: 32492865 PMC7346131

[8] Guillaumet-Adkins A Yañez Y Peris-Diaz MD Calabria I Palanca-Ballester C Sandoval J. Epigenetics and oxidative stress in aging. Oxidative Med Cell Longev. (2017) 2017:1–8. doi: 10.1155/2017/9175806 PMC5541801 28808499

[9] Volpe CMO Villar-Delfino PH dos Anjos PMF Nogueira-Machado JA. Cellular death, reactive oxygen species (ROS) and diabetic complications. Cell Death Dis. (2018) 9:1–9. doi: 10.1038/s41419-017-0135-z 29371661 PMC5833737

[10] Fuentes E Moore-Carrasco R de Andrade Paes AM Trostchansky A. Role of platelet activation and oxidative stress in the evolution of myocardial infarction. J Cardiovasc Pharmacol Ther. (2019) 24:509–20. doi: 10.1177/1074248419861437, PMID: 31280622

[11] Senoner T Dichtl W. Oxidative stress in cardiovascular diseases: still a therapeutic target? Nutrients. (2019) 11:2090. doi: 10.3390/nu11092090, PMID: 31487802 PMC6769522

[12] van der Pol A van Gilst WH Voors AA van der Meer P. Treating oxidative stress in heart failure: past, present and future. Eur J Heart Fail. (2019) 21:425–35. doi: 10.1002/ejhf.1320, PMID: 30338885 PMC6607515

[13] Hayes JD Dinkova-Kostova AT Tew KD. Oxidative stress in cancer. Cancer Cell. (2020) 38:167–97. doi: 10.1016/j.ccell.2020.06.001, PMID: 32649885 PMC7439808

[14] Valko M Rhodes CJ Moncol J Izakovic M Mazur M. Free radicals, metals and antioxidants in oxidative stress-induced cancer. Chem Biol Interact. (2006) 160:1–40. doi: 10.1016/j.cbi.2005.12.009 16430879

[15] Toboła-Wróbel K Pietryga M Dydowicz P Napierała M Brązert J Florek E. Association of oxidative stress on pregnancy. Oxidative Med Cell Longev. (2020) 2020:1–12. doi: 10.1155/2020/6398520 PMC7512072 33014274

[16] Pangrazzi L Balasco L Bozzi Y. Oxidative stress and immune system dysfunction in autism Spectrum disorders. Int J Mol Sci. (2020) 21:3293. doi: 10.3390/ijms21093293, PMID: 32384730 PMC7247582

[17] Corona JC. Role of oxidative stress and neuroinflammation in attention-deficit/hyperactivity disorder. Antioxidants. (2020) 9:1039. doi: 10.3390/antiox9111039, PMID: 33114154 PMC7690797

[18] Chen X Guo C Kong J. Oxidative stress in neurodegenerative diseases. Neural Regen Res. (2012) 7:376–85. doi: 10.3969/j.issn.1673-5374.2012.05.009, PMID: 25774178 PMC4350122

[19] da Fonseca LJS Nunes-Souza V Goulart MOF Rabelo LA. Oxidative stress in rheumatoid arthritis: what the future might hold regarding novel biomarkers and add-on therapies. Oxidative Med Cell Longev. (2019) 2019:1–16. doi: 10.1155/2019/7536805 PMC6942903 31934269

[20] Lu Z Pu C Zhang Y Sun Y Liao Y Kang Z. Oxidative stress and psychiatric disorders: evidence from the bidirectional Mendelian randomization study. Antioxidants. (2022) 11:1386. doi: 10.3390/antiox11071386, PMID: 35883877 PMC9312055

[21] Rapa SF Di Iorio BR Campiglia P Heidland A Marzocco S. Inflammation and oxidative stress in chronic kidney disease—potential therapeutic role of minerals, vitamins and plant-derived metabolites. Int J Mol Sci. (2019) 21:263. doi: 10.3390/ijms21010263, PMID: 31906008 PMC6981831

[22] Cichoż-Lach H Michalak A. Oxidative stress as a crucial factor in liver diseases. World J Gastroenterol: WJG. (2014) 20:8082–91. doi: 10.3748/wjg.v20.i25.8082 25009380 PMC4081679

[23] Hecker L. Mechanisms and consequences of oxidative stress in lung disease: therapeutic implications for an aging populace. Am J Physiol-Lung Cell Mol Physiol. (2018) 314:L642–53. doi: 10.1152/ajplung.00275.2017, PMID: 29351446 PMC5966777

[24] Taha R Blaise GA. Update on the pathogenesis of complex regional pain syndrome: role of oxidative stress. Can J Anaesth J Can Anesth. (2012) 59:875–81. doi: 10.1007/s12630-012-9748-y 22798149

[25] Antwi-Boasiako C Dankwah G Aryee R Hayfron-Benjamin C Donkor E Campbell A. Oxidative profile of patients with sickle cell disease. Med Sci. (2019) 7:17. doi: 10.3390/medsci7020017, PMID: 30691006 PMC6410293

[26] Terrill JR Radley-Crabb HG Iwasaki T Lemckert FA Arthur PG Grounds MD. Oxidative stress and pathology in muscular dystrophies: focus on protein thiol oxidation and dysferlinopathies. FEBS J. (2013) 280:4149–64. doi: 10.1111/febs.12142, PMID: 23332128

[27] Stepien KM Roncaroli F Turton N Hendriksz CJ Roberts M Heaton RA. Mechanisms of mitochondrial dysfunction in lysosomal storage disorders: a review. J Clin Med. (2020) 9:2596. doi: 10.3390/jcm9082596, PMID: 32796538 PMC7463786

[28] Kwon DH Cha HJ Lee H Hong SH Park C Park SH. Protective effect of glutathione against oxidative stress-induced cytotoxicity in RAW 264.7 macrophages through activating the nuclear factor erythroid 2-related factor-2/heme oxygenase-1 pathway. Antioxidants. (2019) 8:82. doi: 10.3390/antiox8040082, PMID: 30939721 PMC6523540

[29] Mori F Natali L Danesi R Nannizzi S Farina C. Post-translational modifications and antioxidant properties of different therapeutic human serum albumins. Int J Biol Macromol. (2021) 183:927–35. doi: 10.1016/j.ijbiomac.2021.05.046, PMID: 33971232

[30] Taverna M Marie AL Mira JP Guidet B. Specific antioxidant properties of human serum albumin. Ann Intensive Care. (2013) 3:4. doi: 10.1186/2110-5820-3-4, PMID: 23414610 PMC3577569

[31] Elliot SJ Catanuto P Pereira-Simon S Xia X Pastar I Thaller S. Catalase, a therapeutic target in the reversal of estrogen-mediated aging. Mol Ther. (2022) 30:947–62. doi: 10.1016/j.ymthe.2021.06.020, PMID: 34174444 PMC8821897

[32] Strehlow K Rotter S Wassmann S Adam O Grohé C Laufs K. Modulation of antioxidant enzyme expression and function by estrogen. Circ Res. (2003) 93:170–7. doi: 10.1161/01.RES.0000082334.17947.11 12816884

[33] Maheshwari S Kumar V Bhadauria G Mishra A. Immunomodulatory potential of phytochemicals and other bioactive compounds of fruits: a review. Food Front. (2022) 3:221–38. doi: 10.1002/fft2.129

[34] Yang L Palliyaguru DL Kensler TW. Frugal chemoprevention: targeting Nrf2 with foods rich in Sulforaphane. Semin Oncol. (2016) 43:146–53. doi: 10.1053/j.seminoncol.2015.09.013, PMID: 26970133 PMC4789124

[35] Nutrient Data Laboratory (U.S.). USDA database for the oxygen radical absorbance capacity (ORAC) of selected foods USDA (2010).

[36] Audousset C McGovern T Martin JG. Role of Nrf2 in disease: novel molecular mechanisms and therapeutic approaches–pulmonary disease/asthma. Front Physiol. (2021) 12:727806. doi: 10.3389/fphys.2021.727806 34658913 PMC8511424

[37] Li M van Esch BCAM Henricks PAJ Garssen J Folkerts G. Time and concentration dependent effects of short chain fatty acids on lipopolysaccharide-or tumor necrosis factor α-induced endothelial activation. Front Pharmacol. (2018) 9:233. doi: 10.3389/fphar.2018.00233, PMID: 29615908 PMC5867315

[38] Dong R Liu S Zheng Y Zhang X He Z Wang Z. Release and metabolism of bound polyphenols from carrot dietary fiber and their potential activity in in vitro digestion and colonic fermentation. Food Funct. (2020) 11:6652–65. doi: 10.1039/D0FO00975J, PMID: 32657286

[39] Carlsen MH Halvorsen BL Holte K Bøhn SK Dragland S Sampson L. The total antioxidant content of more than 3100 foods, beverages, spices, herbs and supplements used worldwide. Nutr J. (2010) 9:3. doi: 10.1186/1475-2891-9-3, PMID: 20096093 PMC2841576

[40] Arreola R Quintero-Fabián S López-Roa RI Flores-Gutiérrez EO Reyes-Grajeda JP Carrera-Quintanar L. Immunomodulation and anti-inflammatory effects of garlic compounds. J Immunol Res. (2015) 2015:401630:1–13. doi: 10.1155/2015/401630 25961060 PMC4417560

[41] Cho HY Reddy SP Kleeberger SR. Nrf2 defends the lung from oxidative stress. Antioxid Redox Signal. (2006) 8:76–87. PMID: 16487040 10.1089/ars.2006.8.76

[42] Guerrero-Beltrán CE Calderón-Oliver M Pedraza-Chaverri J Chirino YI. Protective effect of sulforaphane against oxidative stress: recent advances. Exp Toxicol Pathol Off J Ges Toxikol Pathol. (2012) 64:503–8. doi: 10.1016/j.etp.2010.11.005, PMID: 21129940

[43] Bousquet J Anto JM Czarlewski W Haahtela T Fonseca SC Iaccarino G. Cabbage and fermented vegetables: from death rate heterogeneity in countries to candidates for mitigation strategies of severe COVID-19. Allergy. (2021) 76:735–50. doi: 10.1111/all.14549, PMID: 32762135 PMC7436771

[44] Heiss E Herhaus C Klimo K Bartsch H Gerhäuser C. Nuclear factor κB is a molecular target for Sulforaphane-mediated anti-inflammatory mechanisms. J Biol Chem. (2022) 276:32008–15. doi: 10.1074/jbc.M104794200, PMID: 11410599

[45] Sedlak TW Nucifora LG Koga M Shaffer LS Higgs C Tanaka T. Sulforaphane augments glutathione and influences brain metabolites in human subjects: a clinical pilot study. Mol Neuropsychiatry. (2018) 3:214–22. doi: 10.1159/000487639, PMID: 29888232 PMC5981770

[46] Gan N Wu YC Brunet M Garrido C Chung FL Dai C. Sulforaphane activates heat shock response and enhances proteasome activity through up-regulation of Hsp27. J Biol Chem. (2010) 285:35528–36. doi: 10.1074/jbc.M110.152686, PMID: 20833711 PMC2975177

[47] Meyer M Kesic MJ Clarke J Ho E Simmen RCM Diaz-Sanchez D. Sulforaphane induces SLPI secretion in the nasal mucosa. Respir Med. (2013) 107:472–5. doi: 10.1016/j.rmed.2012.11.006, PMID: 23195333 PMC3640824

[48] Starrett W Blake DJ. Sulforaphane inhibits de novo synthesis of IL-8 and MCP-1 in human epithelial cells generated by cigarette smoke extract. J Immunotoxicol. (2011) 8:150–8. doi: 10.3109/1547691X.2011.558529, PMID: 21401388

[49] Saeedi-Boroujeni A Mahmoudian-Sani MR. Anti-inflammatory potential of quercetin in COVID-19 treatment. J Inflamm. (2021) 18:3. doi: 10.1186/s12950-021-00268-6, PMID: 33509217 PMC7840793

[50] Mohajeri M Horriatkhah E Mohajery R. The effect of glutamine supplementation on serum levels of some inflammatory factors, oxidative stress, and appetite in COVID-19 patients: a case–control study. Inflammopharmacology. (2021) 29:1769–76. doi: 10.1007/s10787-021-00881-0, PMID: 34709541 PMC8552429

[51] Clark A Mach N. The Crosstalk between the Gut Microbiota and Mitochondria during Exercise. Front Physiol. (2022) 8:319. doi: 10.3389/fphys.2017.00319/full PMC5437217 28579962

[52] Pedersen SS Prause M Williams K Barrès R Billestrup N. Butyrate inhibits IL-1β-induced inflammatory gene expression by suppression of NF-κB activity in pancreatic beta cells. J Biol Chem. (2022) 298:102312. doi: 10.1016/j.jbc.2022.102312, PMID: 35921894 PMC9428856

[53] Dahl SM Rolfe V Walton GE Gibson GR. Gut microbial modulation by culinary herbs and spices. Food Chem. (2023) 409:135286. doi: 10.1016/j.foodchem.2022.135286, PMID: 36599291

[54] Lu QY Rasmussen AM Yang J Lee RP Huang J Shao P. Mixed spices at culinary doses have prebiotic effects in healthy adults: a pilot study. Nutrients. (2019) 11:1425. doi: 10.3390/nu11061425, PMID: 31242596 PMC6627368

[55] Lu Q Summanen PH Lee R Huang J Henning SM Heber D. Prebiotic potential and chemical composition of seven culinary spice extracts. J Food Sci. (2017) 82:1807–13. doi: 10.1111/1750-3841.13792, PMID: 28678344 PMC5600121

[56] Percival SS Vanden Heuvel JP Nieves CJ Montero C Migliaccio AJ Meadors J. Bioavailability of herbs and spices in humans as determined by ex vivo inflammatory suppression and DNA strand breaks. J Am Coll Nutr. (2012) 31:288–94. doi: 10.1080/07315724.2012.10720438, PMID: 23378457

[57] Majewski M. *Allium sativum*: facts and myths regarding human health. Rocz Panstw Zakl Hig. (2014) 65:1–8. PMID: 24964572

[58] Aviello G Abenavoli L Borrelli F Capasso R Izzo AA Lembo F. Garlic: empiricism or science? Nat Prod Commun. (2009) 4:1785–96. doi: 10.1177/1934578X0900401231, PMID: 20120123

[59] Crupi P Faienza MF Naeem MY Corbo F Clodoveo ML Muraglia M. Overview of the potential beneficial effects of carotenoids on consumer health and well-being. Antioxidants. (2023) 12:1069. doi: 10.3390/antiox12051069, PMID: 37237935 PMC10215867

[60] Randjelović P Veljković S Stojiljković N Sokolović D Ilić I Laketić D. The beneficial biological properties of salicylic acid. Acta Fac Medicae Naissensis. (2015) 32:259–65. doi: 10.1515/afmnai-2015-0026

[61] Iddir M Brito A Dingeo G Fernandez Del Campo SS Samouda H La Frano MR. Strengthening the immune system and reducing inflammation and oxidative stress through diet and nutrition: considerations during the COVID-19 crisis. Nutrients. (2020) 12:1562. doi: 10.3390/nu12061562, PMID: 32471251 PMC7352291

[62] Mrityunjaya M Pavithra V Neelam R Janhavi P Halami PM Ravindra PV. Immune-boosting, antioxidant and anti-inflammatory food supplements targeting pathogenesis of COVID-19. Front Immunol. (2020) 11:570122. doi: 10.3389/fimmu.2020.570122, PMID: 33117359 PMC7575721

[63] Lammi C Arnoldi A. Food-derived antioxidants and COVID-19. J Food Biochem. (2021) 45:e13557. doi: 10.1111/jfbc.13557, PMID: 33171544

[64] Trujillo-Mayol I Guerra-Valle M Casas-Forero N Sobral MMC Viegas O Alarcón-Enos J. Western dietary pattern antioxidant intakes and oxidative stress: importance during the SARS-CoV-2/COVID-19 pandemic. Adv Nutr. (2021) 12:670–81. doi: 10.1093/advances/nmaa171, PMID: 33439972 PMC7929475

[65] Bouayed J Bohn T. Exogenous antioxidants—double-edged swords in cellular redox state. Oxidative Med Cell Longev. (2010) 3:228–37. doi: 10.4161/oxim.3.4.12858, PMID: 20972369 PMC2952083

[66] Bjelakovic G Nikolova D Gluud C. Antioxidant supplements and mortality. Curr Opin Clin Nutr Metab Care. (2014) 17:40–4. doi: 10.1097/MCO.0000000000000009, PMID: 24241129

[67] Campbell TC. Untold nutrition. Nutr Cancer. (2014) 66:1077–82. doi: 10.1080/01635581.2014.927687, PMID: 25036857

[68] Koekkoek WACK van Zanten ARH. Antioxidant vitamins and trace elements in critical illness. Nutr Clin Pract Off Publ Am Soc Parenter Enter Nutr. (2016) 31:457–74. 10.1177/0884533616653832 27312081

[69] Macpherson H Pipingas A Pase MP. Multivitamin-multimineral supplementation and mortality: a meta-analysis of randomized controlled trials. Am J Clin Nutr. (2013) 97:437–44. doi: 10.3945/ajcn.112.049304, PMID: 23255568

[70] Sadowska-Bartosz I Bartosz G. Effect of antioxidants supplementation on aging and longevity. Biomed Res Int. (2014) 2014:e404680:1–17. doi: 10.1155/2014/404680 PMC3982418 24783202

[71] Ye Y Li J Yuan Z. Effect of antioxidant vitamin supplementation on cardiovascular outcomes: a meta-analysis of randomized controlled trials. PLoS One. (2013) 8:e56803. doi: 10.1371/journal.pone.0056803, PMID: 23437244 PMC3577664

[72] Rychter AM Hryhorowicz S Słomski R Dobrowolska A Krela-Kaźmierczak I. Antioxidant effects of vitamin E and risk of cardiovascular disease in women with obesity – a narrative review. Clin Nutr. (2022) 41:1557–65. doi: 10.1016/j.clnu.2022.04.032, PMID: 35667272

[73] Ferrell M Wang Z Anderson JT Li XS Witkowski M DiDonato JA. A terminal metabolite of niacin promotes vascular inflammation and contributes to cardiovascular disease risk. Nat Med. (2024) 30:424–34. doi: 10.1038/s41591-023-02793-8, PMID: 38374343 PMC11841810

[74] Ornish D Scherwitz LW Billings JH Gould KL Merritt TA Sparler S. Intensive lifestyle changes for reversal of coronary heart disease. JAMA. (1998) 280:2001–7. doi: 10.1001/jama.280.23.2001, PMID: 9863851

[75] Liu RH. Health benefits of fruit and vegetables are from additive and synergistic combinations of phytochemicals. Am J Clin Nutr. (2003) 78:517S–20S. doi: 10.1093/ajcn/78.3.517S, PMID: 12936943

[76] Liu RH. Potential synergy of phytochemicals in cancer prevention: mechanism of action. J Nutr. (2004) 134:3479S–85S. doi: 10.1093/jn/134.12.3479S, PMID: 15570057

[77] Wang S Meckling KA Marcone MF Kakuda Y Tsao R. Synergistic, additive, and antagonistic effects of food mixtures on total antioxidant capacities. J Agric Food Chem. (2011) 59:960–8. doi: 10.1021/jf1040977, PMID: 21222468

[78] Carlson D Wilson C. Redox imbalance theory of disease, the triple oxidant sink, and the antioxidant lifestyle. Int J Dis Reversal Prev. (2024) 6:23. doi: 10.22230/ijdrp.2024v6n1a417

[79] Brunt VE Gioscia-Ryan RA Casso AG VanDongen NS Ziemba BP Sapinsley ZJ. Trimethylamine-N-oxide promotes age-related vascular oxidative stress and endothelial dysfunction in mice and healthy humans. Hypertension. (2020) 76:101–12. doi: 10.1161/HYPERTENSIONAHA.120.14759, PMID: 32520619 PMC7295014

[80] Zhenyukh O Civantos E Ruiz-Ortega M Sánchez MS Vázquez C Peiró C. High concentration of branched-chain amino acids promotes oxidative stress, inflammation and migration of human peripheral blood mononuclear cells via mTORC1 activation. Free Radic Biol Med. (2017) 104:165–77. doi: 10.1016/j.freeradbiomed.2017.01.009, PMID: 28089725

[81] Gupta P Thompson BL Wahlang B Jordan CT Hilt JZ Hennig B. The environmental pollutant, polychlorinated biphenyls, and cardiovascular disease: a potential target for antioxidant nanotherapeutics. Drug Deliv Transl Res. (2018) 8:740–59. doi: 10.1007/s13346-017-0429-9 28975503 PMC5882597

[82] Gomez-Cabrera M Domenech E Viña J. Moderate exercise is an antioxidant: upregulation of antioxidant genes by training. Free Radic Biol Med. (2008) 44:126–31. doi: 10.1016/j.freeradbiomed.2007.02.001, PMID: 18191748

[83] Vaccaro A Dor YK Nambara K Pollina EA Lin C Greenberg ME. Sleep loss can cause death through accumulation of reactive oxygen species in the gut. Cell. (2020) 181:1307–1328.e15. doi: 10.1016/j.cell.2020.04.049, PMID: 32502393

[84] França MB Panek AD Eleutherio ECA. Oxidative stress and its effects during dehydration. Comp Biochem Physiol A Mol Integr Physiol. (2007) 146:621–31. doi: 10.1016/j.cbpa.2006.02.030 16580854

[85] Sepidarkish M Farsi F Akbari-Fakhrabadi M Namazi N Almasi-Hashiani A Maleki Hagiagha A. The effect of vitamin D supplementation on oxidative stress parameters: a systematic review and meta-analysis of clinical trials. Pharmacol Res. (2019) 139:141–52. doi: 10.1016/j.phrs.2018.11.011, PMID: 30447293

[86] Li H Xia N. The role of oxidative stress in cardiovascular disease caused by social isolation and loneliness. Redox Biol. (2020) 37:101585. doi: 10.1016/j.redox.2020.101585, PMID: 32709420 PMC7767744

[87] Valtorta NK Kanaan M Gilbody S Ronzi S Hanratty B. Loneliness and social isolation as risk factors for coronary heart disease and stroke: systematic review and meta-analysis of longitudinal observational studies. Heart. (2016) 102:1009–16. doi: 10.1136/heartjnl-2015-308790 27091846 PMC4941172

[88] Kamceva G Arsova-Sarafinovska Z Ruskovska T Zdravkovska M Kamceva-Panova L Stikova E. Cigarette smoking and oxidative stress in patients with coronary artery disease. Open Access Maced J Med Sci. (2016) 4:636–40. doi: 10.3889/oamjms.2016.117 28028404 PMC5175512

[89] Tsermpini EE Plemenitaš Ilješ A Dolžan V. Alcohol-induced oxidative stress and the role of antioxidants in alcohol use disorder: a systematic review. Antioxidants. (2022) 11:1374. doi: 10.3390/antiox11071374, PMID: 35883865 PMC9311529

[90] Chainy GBN Sahoo DK. Hormones and oxidative stress: an overview. Free Radic Res. (2020) 54:1–26. doi: 10.1080/10715762.2019.1702656 31868060

[91] Schiavone S Jaquet V Trabace L Krause KH. Severe life stress and oxidative stress in the brain: from animal models to human pathology. Antioxid Redox Signal. (2013) 18:1475–90. doi: 10.1089/ars.2012.4720, PMID: 22746161 PMC3603496

[92] Lettieri-Barbato D Minopoli G Caggiano R Izzo R Santillo M Aquilano K. Fasting drives Nrf2-related antioxidant response in skeletal muscle. Int J Mol Sci. (2020) 21:7780. doi: 10.3390/ijms21207780, PMID: 33096672 PMC7589317

[93] Calabrese V Cornelius C Dinkova-Kostova AT Calabrese EJ Mattson MP. Cellular stress responses, the Hormesis paradigm, and Vitagenes: novel targets for therapeutic intervention in neurodegenerative disorders. Antioxid Redox Signal. (2010) 13:1763–811. doi: 10.1089/ars.2009.3074, PMID: 20446769 PMC2966482

[94] Gómez-Sámano MÁ Grajales-Gómez M Zuarth-Vázquez JM MaF N-F Martínez-Saavedra M Juárez-León ÓA. Fibroblast growth factor 21 and its novel association with oxidative stress. Redox Biol. (2016) 11:335–41. doi: 10.1016/j.redox.2016.12.024 28039838 PMC5200873

[95] Tezze C Romanello V Sandri M. FGF21 as modulator of metabolism in health and disease. Front Physiol. (2019) 10:419. doi: 10.3389/fphys.2019.00419 31057418 PMC6478891

[96] Wang J Yang X Zhang J. Bridges between mitochondrial oxidative stress, ER stress and mTOR signaling in pancreatic β cells. Cell Signal. (2016) 28:1099–104. doi: 10.1016/j.cellsig.2016.05.007 27185188

[97] Gao X Yu X Zhang C Wang Y Sun Y Sun H. Telomeres and mitochondrial metabolism: implications for cellular senescence and age-related diseases. Stem Cell Rev Rep. (2022) 18:2315–27. doi: 10.1007/s12015-022-10370-8, PMID: 35460064 PMC9033418

[98] Gavia-García G Rosado-Pérez J Arista-Ugalde TL Aguiñiga-Sánchez I Santiago-Osorio E Mendoza-Núñez VM. Telomere length and oxidative stress and its relation with metabolic syndrome components in the aging. Biology. (2021) 10:253. doi: 10.3390/biology10040253, PMID: 33804844 PMC8063797

